# C-Reactive Protein Knockout Attenuates Temporomandibular Joint Inflammation in Rats

**DOI:** 10.1155/2022/8613986

**Published:** 2022-01-10

**Authors:** Yao He, Mengjiao Zhou, Zixiang Jian, Lingli Fang, Lan Huang, Jinlin Song

**Affiliations:** ^1^Department of Orthodontics, Stomatological Hospital of Chongqing Medical University, Chongqing, China; ^2^Chongqing Key Laboratory of Oral Disease and Biomedical Sciences, Stomatological Hospital of Chongqing Medical University, Chongqing, China; ^3^Chongqing Municipal Key Laboratory of Oral Biomedical Engineering of Higher Education, Stomatological Hospital of Chongqing Medical University, Chongqing, China

## Abstract

**Background:**

C-reactive protein (CRP), a biomarker of inflammation, is highly expressed in osteoarthritis- (OA-) related diseases, but its exact role remains unknown. In this study, we evaluated the biological effect of CRP on temporomandibular joint (TMJ) inflammation.

**Methods:**

Freund's complete adjuvant (CFA) was used to induce TMJ inflammation in CRP-knockout (CRP-/-) and control rats. Degenerative changes in the TMJ were compared to elucidate the role of CRP in TMJ inflammation. In addition, inflammatory cytokines, macrophage activation, and osteoclast differentiation were evaluated by real-time quantitative polymerase chain reaction, immunohistochemistry, and tartrate-resistant phosphatase staining to explore the potential regulatory mechanism.

**Results:**

Compared to the control, CFA induced TMJ inflammation, which increased systemic and local CRP expression. Furthermore, CRP-/- rats exhibited less severe inflammatory symptoms. The downregulation of proinflammatory cytokines (interleukin- (IL-) 1*β* and IL-6) and upregulation of the anti-inflammatory cytokine IL-10 were detected in CRP-/- rats, which also exhibited reduced macrophage activation and osteoclast differentiation.

**Conclusion:**

These results indicated that controlling the highly elevated levels of CRP during inflammation could modify the cytokine profile, macrophage activation, and osteoclast differentiation, thus, providing beneficial effects for TMJ-OA prevention and treatment.

## 1. Introduction

The temporomandibular joint (TMJ) plays a significant role in daily activities, such as eating, speaking, and facial expression. The mandibular condyle, an interposed fibrocartilaginous disc, and glenoid fossa compose this exquisite joint [1]. As the fourth most common type of disease in stomatology, TMJ disorders (TMDs) affect 5 to 12% of the population, and 65% of patients with rheumatoid arthritis (RA) have TMJ symptoms [2, 3]. Once TMJ diseases progress to TMJ osteoarthritis (TMJ-OA), severe pain and dysfunction occur, which cause heavy burdens in daily life [4].

TMJ-OA is characterized by synovitis, morphological changes in TMJ discs, degeneration of cartilage, and remodelling of subchondral bone [5]. During the progression of TMJ-OA, the synovial membrane is frequently the primary tissue affected by inflammation and undergoes pathological changes, including synovial hyperplasia, increased vascularity, and inflammatory cell infiltration [6]. The TMJ disc, which undergoes morphological changes, becomes thickened, displaced, lengthened, and perforated in the severe stage of disease progression [7]. Additionally, typical degenerative lesions of the mandibular condylar cartilage, including chondrocyte alignment irregularities, nested proliferation and hyalinization, as well as clefts and erosions occur when the disease progresses to a severe stage [8]. Subchondral bone always exhibits resorption, which is accompanied by decreased bone mineral density and increased trabecular thickness, bone sclerosis, and osteophyte formation [9].

Inflammation is believed to be a vital factor in the pathological procession of TMJ-OA, since proinflammatory cytokines, such as tumour necrosis factor-alpha (TNF-*α*) and interleukin- (IL-) 1*β* and IL-6, are elevated in the synovial fluid of TMD patients [6, 10]. The upregulation of inflammatory factors exacerbates the development of TMJ-OA, and medical treatments that target inflammatory cytokines and the related signalling pathways show beneficial effects on the resolution of inflammation and recovery from TMJ-OA [11, 12]. TNF-*α* inhibitors and IL-1 receptor antagonists are well-established therapeutic approaches for RA treatment [13–15], and antibodies against TNF-*α*, IL-1*β*, and IL-6 show positive effects during investigation of RA therapies [16, 17]. Thus, treatments that control inflammation, especially treatments focused on reducing inflammatory factors, play an important role in TMJ-OA therapy.

C-reactive protein (CRP), a reactive protein of the acute phase of inflammation, can be induced by inflammatory cytokines (TNF-*α*, IL-1*β*, and IL-6), synthesized by hepatocytes and released into the circulatory system [18]. Although it has long been regarded as a marker of acute inflammation, recent studies have shown that CRP also plays an important regulatory role in a variety of chronic inflammatory diseases [19, 20]. It has long been recognized that elevated serum CRP levels positively correlate with RA severity and progression, and systemic CRP levels are significantly increased in OA patients [21, 22]. Several clinical studies have also confirmed that increased serum CRP levels are associated with TMJ pain and bone loss in patients [23, 24].

Despite all these associations, the exact biological role of CRP in OA remains unknown. No clinical research has directly revealed the effect of CRP on OA, and to date, animal studies have yielded mixed results [25]. CRP expression aggravated bone erosion in an OA rat model and promoted osteoclastogenesis in vitro, demonstrating a detrimental role of CRP in OA [25, 26]. However, a previous study showed that collagen-induced arthritis (CIA) was exacerbated in CRP-deficient mice, and the transgenic expression of human CRP inhibited the development of OA, suggesting a protective effect of CRP [27, 28]. The exact role of CRP in TMJ-OA has not yet been determined. In this study, CRP-knockout (CRP-/-) rats were generated and developed by the transcription activator-like effector nuclease (TALEN) technique. Freund's complete adjuvant (CFA) was used to induce TMJ inflammation in CRP-/- and control rats. Degenerative changes in the TMJs were compared to elucidate the exact role of CRP in TMJ inflammation. Moreover, inflammatory cytokines, macrophage activation, and osteoclast differentiation were evaluated to explore the potential regulatory mechanism.

## 2. Materials and Methods

The experiments were conducted following protocols approved by the Animal Ethics Committee at Chongqing Medical University (AECCMU-2020-004). All the methods were in accordance with the approved guidelines.

### 2.1. Animals and Induction of TMJ Inflammation

Adult female Sprague Dawley (SD) rats aged 10-12 weeks and weighing 350-400 g were used as controls for CRP-/- rats of the same age and weight. The CRP-/- rats were provided by Professor Zhigang Yang from Chongqing Medical School [29]. DNA sequencing was performed by Sangon Biotech (Shanghai, China) to determine the knockout efficiency in CRP-/- rats. The animals were housed in the Experimental Animal Center of Stomatological Hospital of Chongqing Medical University and maintained in a barrier room at 25°C with 40% humidity and a 12-hour light/dark cycle, and the animals were given free access to food and water.

Inflammation was induced by injection of 50 *μ*l CFA (Sigma, MO, USA) into the upper compartment of the bilateral TMJ [30]. The rats in which inflammation was not induced received injections of the same volume of saline. Thirty-two rats were included and divided into 4 groups. The control group consisted of SD rats that received saline injection, and the CFA group consisted of rats that received CFA injection. The CRP-/- rats injected with saline were classified as the CRP group, while those injected with CFA were classified as the CRP + CFA (*C* + *C*) group.

### 2.2. Tissue Harvest and Disc Weight Measurement

The rats were sacrificed by carbon dioxide inhalation 7 days after inflammation induction. The width of the heads from one side of the TMJ to the other side was measured to indicate the swelling of the TMJ areas after CFA induction according to Wang et al.'s study [7]. After the heads were hemisected, one side of the TMJ capsules was opened, the naked condyles were exposed, and the disc tissue was separated. The contralateral side of the TMJ was not disarticulated but fixed *in situ* in 4% paraformaldehyde (PFA, Affymetrix, USA) at 4°C for slide sectioning. The separated discs were carefully cleaned and weighed after the excess water was removed. The discs and synovia from the same rat were then separated and collected for PCR analysis.

### 2.3. Paraffin Section Preparation and Staining

The PFA-fixed hemisected heads were decalcified with 14% ethylenediaminetetraacetic acid (EDTA; pH 7.5) for up to 2 months. After gradient decalcification, paraffin was used to embed the samples. Sagittal sections (5 *μ*m thick) were obtained before staining with haematoxylin and eosin (H&E), safranin O and fast green (Solarbio, China) to observe the histological changes in the TMJ using standard methods. Tartrate-resistant phosphatase (TRAP) staining (Solarbio, China) was performed to estimate the osteoclast activity. Sections from equivalent regions of the TMJ were compared between animals.

### 2.4. Histological Analysis

Slides stained with H&E were used for histological analysis. Disc thickness measurements were performed in the anterior, intermediate, and posterior bands of 6 randomly selected sections from every joint (*N* = 6/group) according to the method described in Wang et al.'s study [7]. The measurements were performed by two investigators after professional training from a TMJ specialist. The extended lines of the intermediate band in the cartilage were gauged to determine the thickness of the total cartilage, fibrocartilage layers, and hypertrophic chondrocyte layers with safranin O and fast green staining.

The inflammatory score of the synovial membrane was semiquantitatively evaluated with the following parameters. One scale was used to assess for inflammatory infiltration: no infiltration = 0, discrete infiltration = 1, moderate infiltration = 2, and intense infiltration = 3. Another scale was used to assess synovial membrane thickness: no thickening = 0, discrete thickening = 1, moderate thickening = 2, and intense thickening = 3 [8]. The total number of mononucleated cells in the synovial membrane of a 100 × 100 *μ*m square was counted for inflammatory cell infiltration measurement (*N* = 6/group, 6 randomly selected sections per joint).

### 2.5. Immunohistochemistry

Immunohistochemical staining was performed with sections that had been deparaffinized and rehydrated. Slides were incubated with 0.3% hydrogen peroxide for 20 min before incubation with serum to reduce nonspecific binding. The slides were then incubated overnight with anti-CRP 1 : 200 (Abcam, USA), anti-IL-1*β* 1 : 100 (Bioss, China), anti-IL-10 1 : 150 (Bioss, China), anti-TNF-*α* 1 : 100 (Bioss, China), anti-CD68 1 : 100 (Abcam, USA), anti-inducible nitric oxide synthase (iNOS) 1 : 100 (Affinity, USA), and antireceptor activator of NF-*κ*B (RANKL) 1 : 100 (Bioss, China). Then, the slides were washed and incubated with goat anti-mouse secondary antibodies for 30 min (Zhongshan Biotechnology, China), visualized with 3,3-diaminobenzidine tetrahydrochloride (DAB) substrate (Zhongshan Biotechnology, China), and counterstained with haematoxylin. Image-Pro Plus (6.0, Media Cybernetics, Rockville, MD) was used for the semiquantitative analysis, and protein expression was evaluated based on the integrated optical density (IOD).

### 2.6. Real-Time Quantitative Polymerase Chain Reaction (RT-qPCR)

Total RNA was isolated from discs and synovia with TRIzol reagent (Invitrogen, USA) following the instructions. RNA qualification, reverse transcription, and polymerase chain reaction were performed as previously described in detail [31]. The sequences of the commercially synthesized primers are listed below.

### 2.7. Enzyme-Linked Immunosorbent Assay (ELISA)

High-sensitivity CRP (hsCRP) levels were quantified with a commercially available ELISA kits (Zhenke, China) based on the manufacturer's instruction. After centrifuging the blood from the rats, the serum was acquired and diluted 4000 times with double-distilled water. The diluted samples were added to each well of an enzyme label plate and incubated at 25°C for 30 min before washing 5 times. Then, 100 *μ*l of substrate was added into the wells, and the colour was developed in the dark at 25°C for 10 min. Then, 100 *μ*l of termination solution was added to each well. The optical density was measured at 405 nm with 650 nm wavelength correction.

### 2.8. Western Blot Analysis

Proteins were extracted from hepatic tissue using radioimmunoprecipitation assay (RIPA) lysis buffer (Thermo Fisher Scientific, USA). The total proteins were separated and transferred to polyvinylidene fluoride membranes. The membranes were blocked with 5% bovine serum albumin (BSA) in phosphate-buffered saline (PBS) for 60 min at room temperature and then probed with anti-CRP (1 : 1000; Abcam, USA) or anti-*β*-actin (1 : 5000; Zhengneng, China) at 4°C overnight. Then, after incubation with goat anti-rabbit IgG H&L secondary antibodies (1 : 500; Abcam, USA) for 60 min, the immunoreactive proteins were detected with a chemiluminescence kit (Millipore, USA) by enhanced chemiluminescence (Amersham Pharmacia Biotech, USA).

### 2.9. Statistical Analysis

SPSS 23.0 software (SPSS Inc., USA) was used for the data analysis. The data are expressed as the mean + standard deviation (SD). The normality of the data distribution was examined by the Shapiro-Wilk test, and Levene's test was used to assess the homogeneity of variance. Statistical comparisons were performed using independent *t*-tests (two-group comparisons) or one-way analysis of variance (ANOVA; more than two-group comparisons) followed by multiple comparisons using Tukey's test. Statistical significance was defined as *p* < 0.05.

## 3. Results

### 3.1. CFA Induced TMJ Inflammation with CRP Overexpression

**Figure 1:**
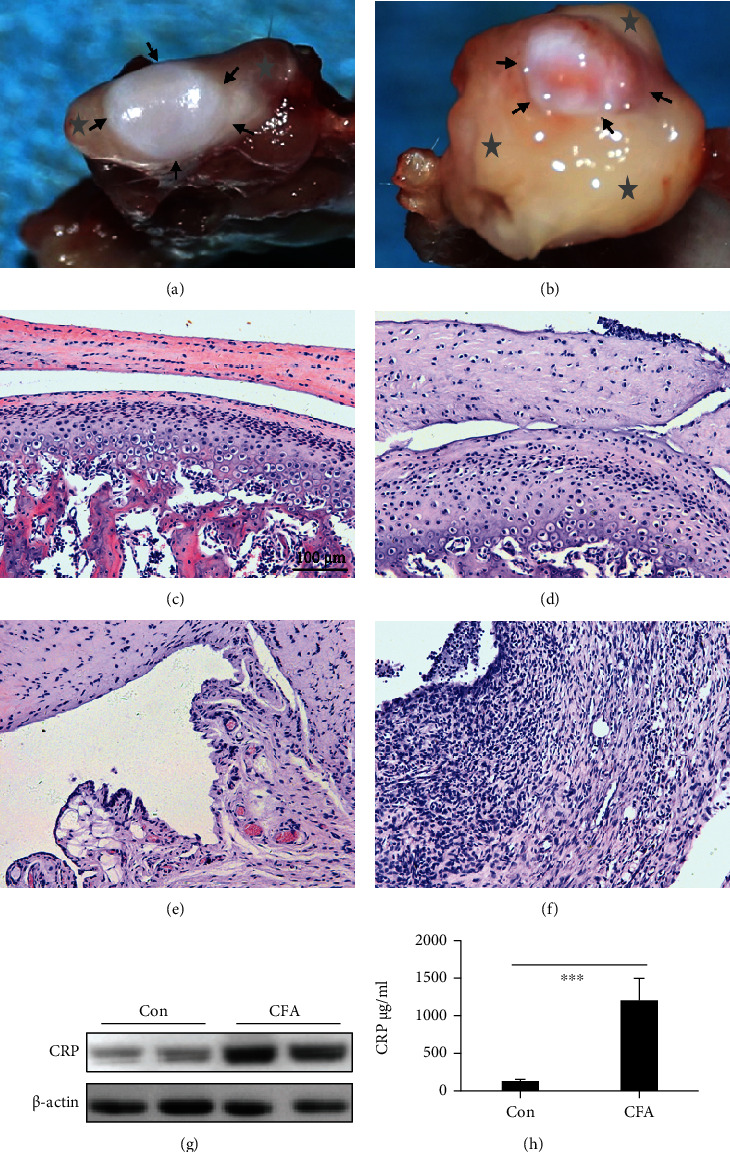
CFA induced TMJ inflammation with CRP overexpression. (a) and (b) Representative images of exposed TMJs and swollen and hyperplastic synovial membranes in the control and CFA groups, respectively. The part around with arrows is disc, and synovium is covered with grey stars. (c) and (d) Representative images of H&E-stained slides show the middle part of the TMJ disc and cartilage of control and CFA groups, respectively. (e) and (f) Images of H&E-stained slides showing synovial membranes from the anterior superior region of control and CFA groups, respectively. (g) CRP protein expression of hepatic tissue (each protein with 2 parallels). (h) Serum CRP level between control and CFA group (*N* = 4; mean + SD; ∗∗∗*p* < 0.001).

According to a study on the CFA-induced, time-dependent degeneration of the TMJ in rats, obvious inflammatory symptoms appeared 7 days after injection, and the swelling gradually subsided after that time point [7]. We chose to collect samples 7 days after CFA induction. In the CFA group, swollen TMJs and excessive synovial hyperplasia were readily apparent (Figures 1(a) and 1(b)). Thickened discs, chondrocyte alignment irregularities, and inflammatory cell infiltration were also observed in the CFA group (Figures 1(c)–1(f)). Moreover, hepatic CRP expression and serum CRP levels were distinctly elevated in the CFA group (Figures 1(g) and 1(h)).

### 3.2. Inflammatory Manifestations in the TMJ of CRP-Knockout Rats

**Figure 2:**
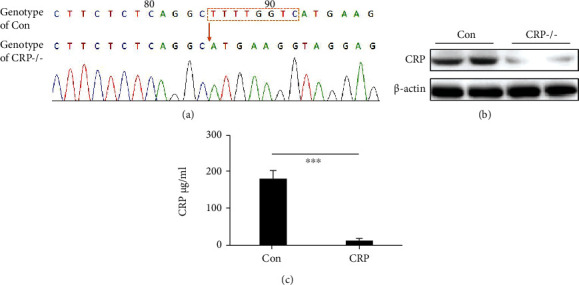
Identification of CRP knockout in rats. (a) Sequencing chromatogram of CRP -/- rats. The bases in orange box missed in CRP -/- rats. (b) CRP protein expression of hepatic tissue (each protein with 2 parallels). (c) Serum CRP level between control and CRP group (*N* = 4; mean + SD; ∗∗∗*p* < 0.001).

On the premise that CFA-induced TMJ inflammation increases CRP expression, we used CRP-/- rats to explore the effect of CRP on TMJ inflammation. The DNA sequencing results for these rats showed deletions in numerous exons. In addition, CRP-/- rats showed decreased CRP protein expression in hepatic tissue and reduced serum CRP levels (Figure 2).

**Figure 3:**
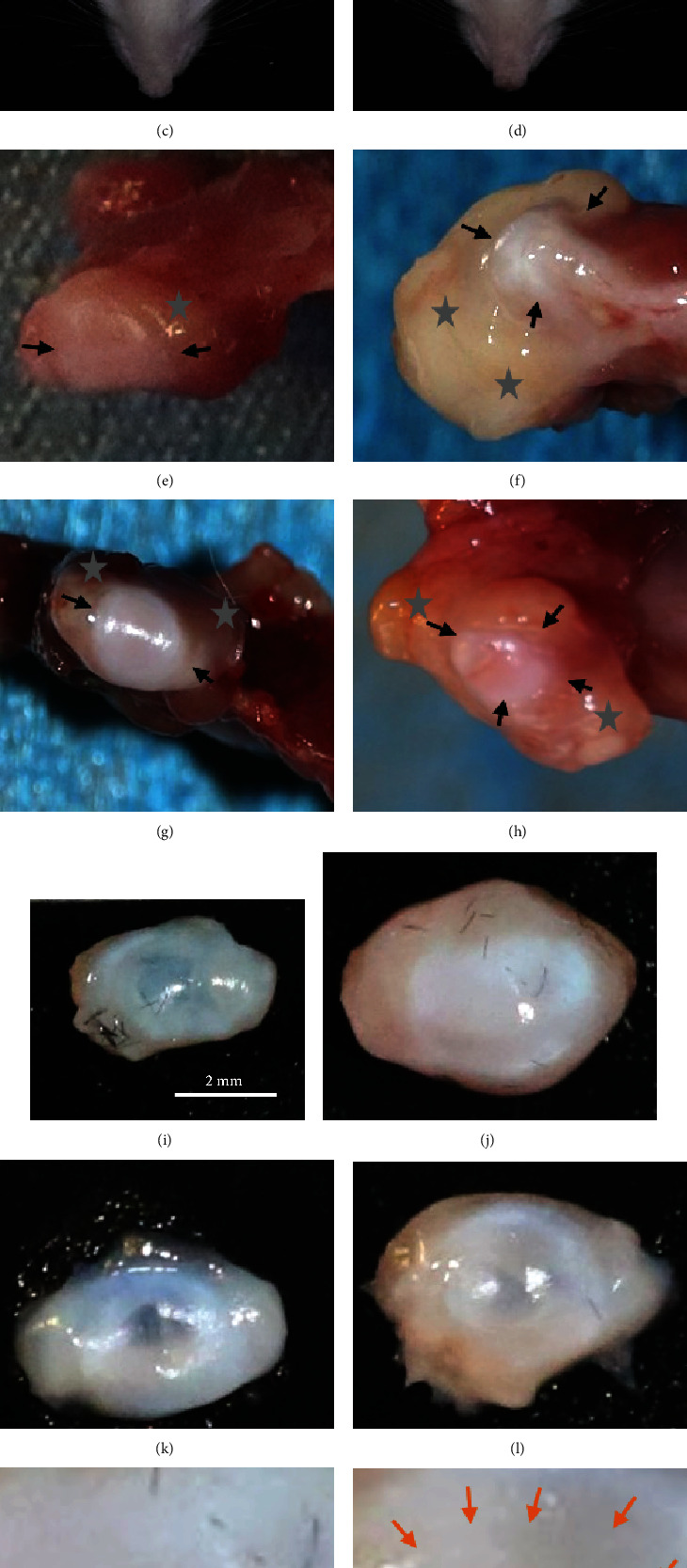
Inflammatory manifestations in rat TMJs. (a)–(d) Representative images of rat heads with linear head width marked as the distance between the bilateral TMJs from Con, CFA, CRP, and *C* + *C* groups, respectively. (e)–(h) Representative images of exposed TMJs and synovial membranes from Con, CFA, CRP, and *C* + *C* groups, respectively. The part around with arrows is disc, and synovium covered with grey stars. Note swollen and hyperplastic synovial membranes in the CFA and *C* + *C* groups. (i)–(l) Representative images of discs from Con, CFA, CRP, and *C* + *C* groups, respectively; the thickened opaque discs from the CFA group were obvious. (m) and (n) Magnified images of (j) and (l) showed central middle of disc, which should be transparent in normal (area around with arrows in (n)). Note that this area disappeared in the CFA group (m). (o) Different of head widths among groups. Note the width increase in *C* + *C* group is not that much in the CFA group compared to their corresponding control groups. (p) Net weights of the discs showing significantly higher weights in the inflammation groups than in the control groups (*N* = 8; mean + SD; ∗∗∗*p* < 0.001); Con: control, *C* + *C*: CRP + CFA group.

A marked increase in the linear head width between the bilateral TMJs in the CFA and CRP + CFA groups was observed, and this increase demonstrated swelling of the TMJ areas (Figures 3(a)–3(d) and 3(o)). When the articular cavity was exposed, swollen and hyperplastic synovial membranes (Figures 3(e)–3(h)) and thick opacity discs (Figure 3(i)–3(n)) were observed in the CFA group. Compared to those in the CFA group, the inflammatory changes in the *C* + *C* group seemed milder (Figure 3(p)).

### 3.3. Tissue, Cellular, and Molecular Changes in the Inflamed TMJ of CRP-Knockout Rats

**Figure 4:**
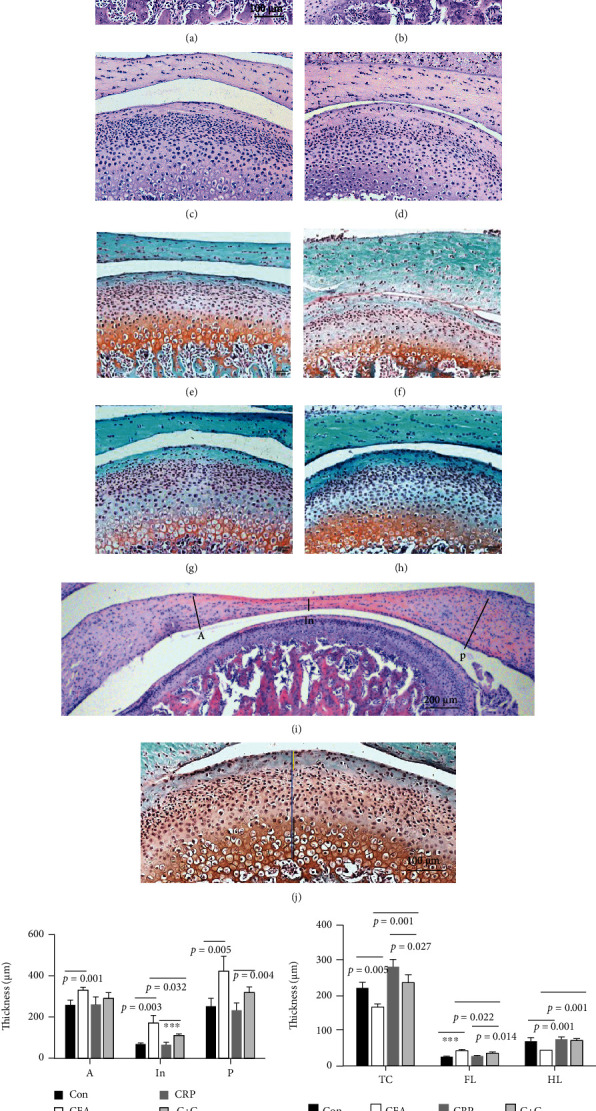
Histological changes in the TMJ disc and cartilage. (a)–(d) Representative images of H&E-stained slides show the middle part of the TMJ disc and cartilage from Con, CFA, CRP, and *C* + *C* groups, respectively. (e)–(h) Representative images of safranin O- and fast green-stained slides involving TMJ discs and cartilage from Con, CFA, CRP, and *C* + *C* groups, respectively. (i) Photomicrograph showing the disc thickness measurement method (A: anterior band; In: intermediate zone; P: posterior band). (j) Photomicrograph of cartilage thickness measurement, with a blue line indicating the total cartilage thickness (TC), a yellow line indicating the fibrocartilage layer thickness (FL), and a black line indicating the hypertrophic chondrocyte layer thickness (HL). (k) Statistical results of disc thickness in every group. (l) Statistical results of cartilage thickness in every group. (*N* = 6; mean + SD; ∗∗∗*p* < 0.001).

The H&E slides of discs and cartilage showed thickened and deformed discs in the CFA group. In addition, abnormal performance of the condylar cartilage was revealed in the CFA group with more dispersed cells and an irregular organization compared to the control group, and the cartilage surface seemed to be adhered to some areas of the articular disc tissue. In the *C* + *C* group, only thickened articular discs were observed, and changes in condylar cartilage were not obvious compared to those in the CRP and control groups (Figures 4(a)–4(h)). According to the measurement of the anterior, intermediate, and posterior bands, the whole disc was thickened after CFA induction. The decline of disc thickness in the intermediate zone was obvious in the *C* + *C* group compared to the CFA group. Although a decline was observed, no significant differences of disc thickness were observed when CRP was deleted in the anterior and posterior zones after CFA induction (Figures 4(i) and 4(k)).

With safranin O and fast green staining, a decline in the total cartilage thickness was observed in the CFA group, which was caused by a decrease in the hypertrophic chondrocyte layers, while the thickness of the fibrocartilage layers was significantly increased in the CFA group compared to the control group. The differences between the CRP and *C* + *C* groups were not that obvious, but increased fibrocartilage layers were observed in the *C* + *C* group (Figures 4(e)–4(h), 4(j), and 4(l)).

**Figure 5:**
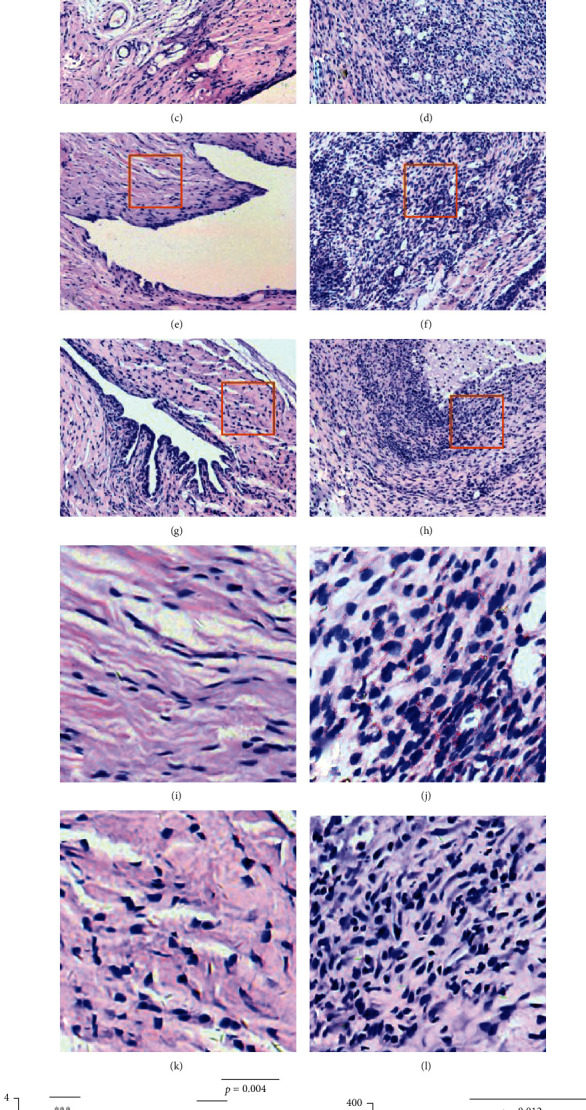
Histological changes in the synovial membrane. (a)–(d) Representative images of H&E-stained slides showing synovial membranes of the anterior superior region from Con, CFA, CRP, and *C* + *C* groups, respectively. (e)–(h) Representative images of synovial membranes of the posterior superior region from Con, CFA, CRP, and *C* + *C* groups, respectively. The orange box indicates a 100 × 100 *μ*m square used for counting infiltrating inflammatory cells. (i)–(l) Magnified images of areas in orange boxes from (e)–(h). (m) Inflammatory score based on inflammatory cell infiltrate (II) and thickening of the synovial membrane (TS). (n) Number of mononucleated cells in a 100 × 100 *μ*m square of the synovial membrane measured to determine inflammatory infiltration. (*N* = 6; mean + SD; ∗∗∗*p* < 0.001).

Compared with the disc and cartilage, the synovial membrane was the most sensitive tissue for the induction of inflammation. In part, because CFA was injected into the superior articular cavity, the synovial membrane in the upper compartment experienced more severe inflammation (Figures 5(a)–5(l)). Several characteristic changes in inflammation were easily identified in the CFA and *C* + *C* groups, and these changes included the apparent infiltration of mononucleated cells, marked proliferation of lining cells, and abundant lipid droplets in the synovium, and the CFA group showed more apparent changes than the *C* + *C* group (Figure 5).

### 3.4. CRP Altered the Cytokine Profile in the Disc and Synovial Membranes of the Inflamed TMJ

**Figure 6:**
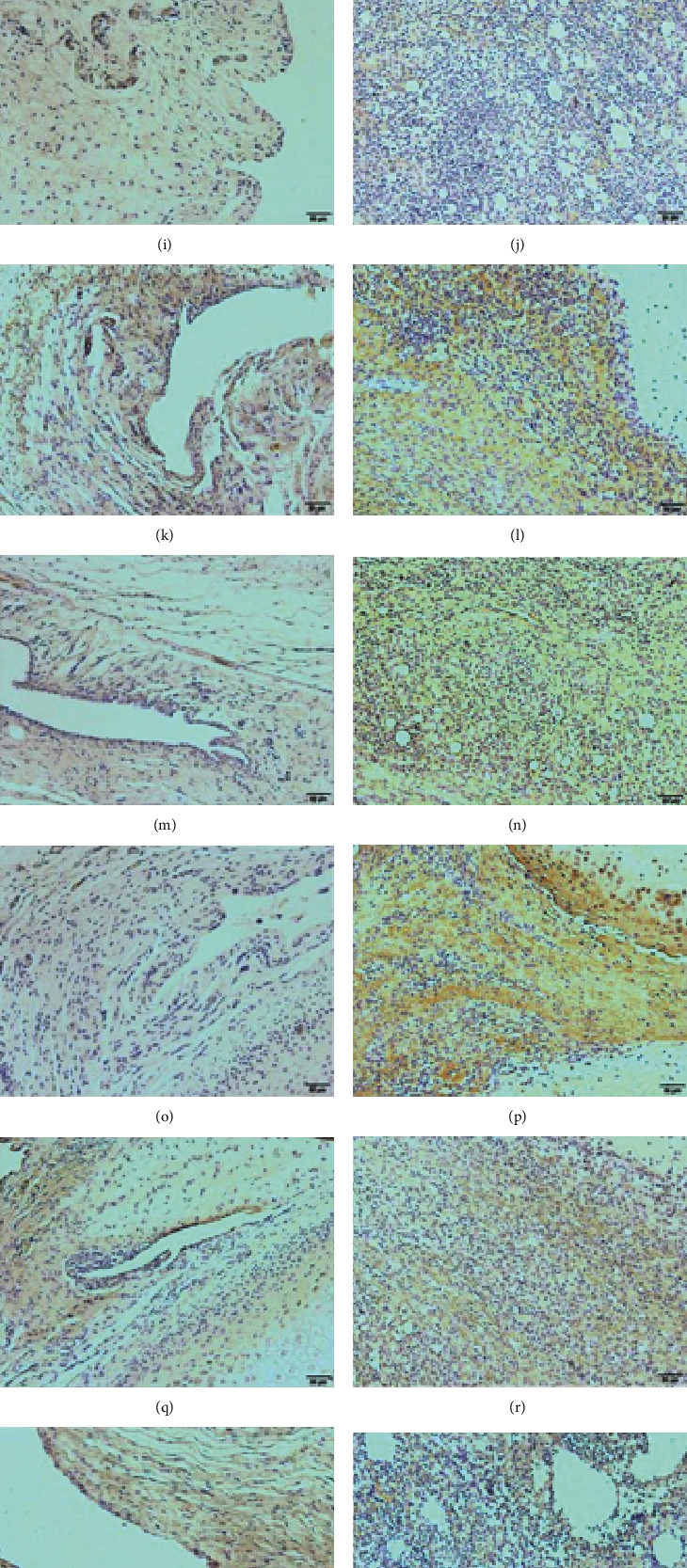
Inflammatory cytokine expression based on RT-qPCR and IHC results. The mRNA expression levels of (a) CRP, (b) TNF-*α*, (c) IL-1*β*, (d) IL-2, (e) IL-6, and (f) IL-10. (*N* = 8; mean + SD; ∗∗∗*p* < 0.001). (g)–(j) The IHC results of CRP expression in the synovial membrane from Con, CFA, CRP, and *C* + *C* groups, respectively. (k)–(n) The IHC results of TNF-*α* from Con, CFA, CRP, and *C* + *C* groups, respectively. (o)–(r) The IHC results of IL-1*β* from Con, CFA, CRP, and *C* + *C* groups, respectively. (s)–(v) The IHC results of IL-10 from Con, CFA, CRP, and *C* + *C* groups, respectively. (w)–(z) Semiquantitative analysis of (g)–(v). (*N* = 6; mean + SD; ∗∗∗*p* < 0.001).

CRP mRNA levels increased after CFA induction (Figure 6(a)). The inflammatory cytokines IL-1*β* and IL-6 were at higher levels in the CFA group than in the *C* + *C* group. Moreover, IL-10, which is considered an anti-inflammatory factor, was distinctly decreased in the CFA group. When CRP was knocked out, IL-10 expression increased (Figures 6(c), 6(e), and 6(f)). Elevated expression of TNF-*α* and IL-2 was found in the CFA and *C* + *C* groups, but the difference between these two groups was not significant (Figures 6(b) and 6(d)). The IHC results of CRP, IL-1*β*, and IL-10 revealed that the protein expression trends were similar to the mRNA levels, but more TNF-*α* expression was detected in the CFA group than in the *C* + *C* group (Figures 6(g)–6(z)).

### 3.5. CRP Affected Macrophage and Osteoclast Activity in TMJ Inflammation

**Figure 7:**
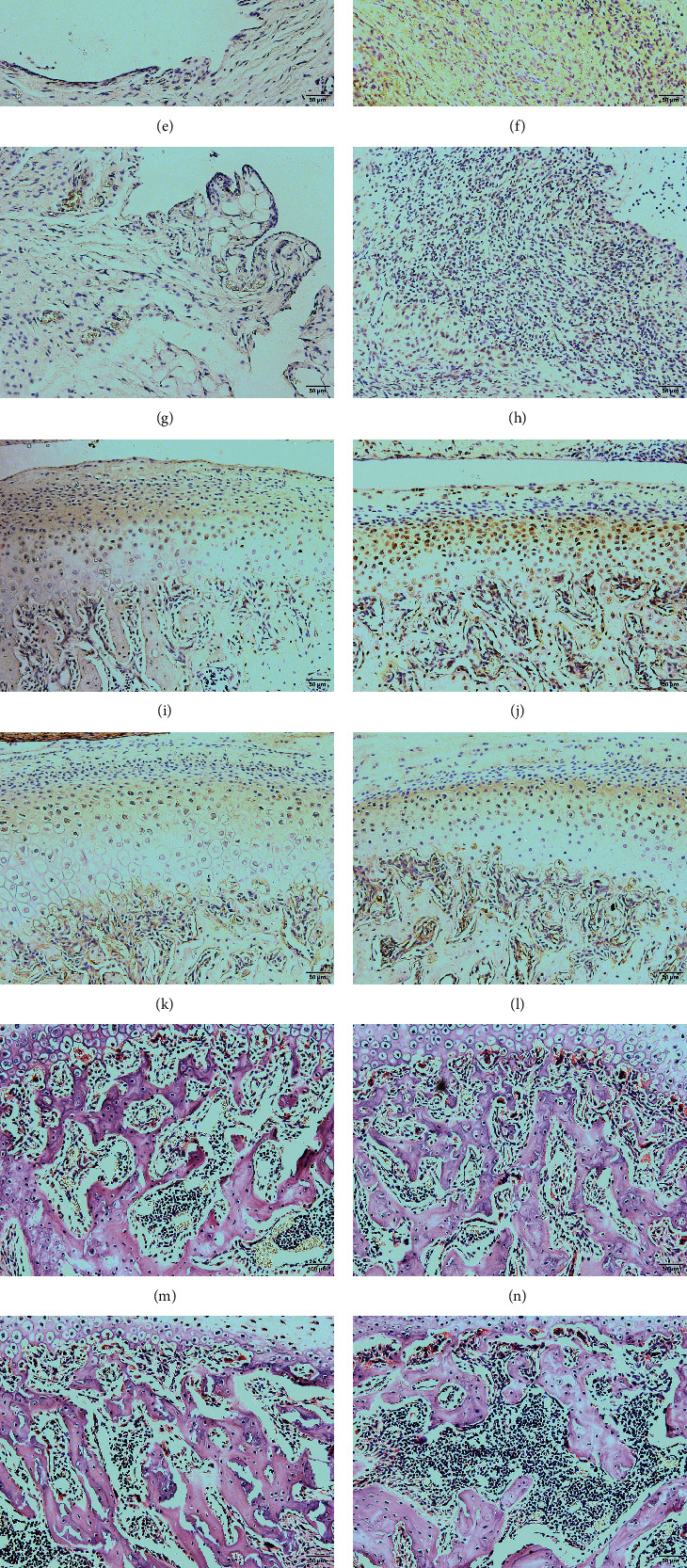
Macrophage and osteoclast activities and photomicrographs of immunopositive cells in the synovium and cartilage. (a)–(d) The IHC results of CD68 expression in the synovial membrane from Con, CFA, CRP, and *C* + *C* groups, respectively. (e)–(h) The IHC results of iNOS from Con, CFA, CRP, and *C* + *C* groups, respectively. (i)–(l) The IHC results of RANKL expression in the TMJ cartilage from Con, CFA, CRP, and *C* + *C* groups, respectively. (m)–(p) TRAP staining of subchondral bone from Con, CFA, CRP, and *C* + *C* groups, respectively. (q)–(s) Semiquantitative analysis of (a)–(l). (t) Statistical results of TRAP-positive cells (clusters of more than 3 nuclei were counted as one osteoclast) in the different groups. (*N* = 6; mean + SD; ∗∗∗*p* < 0.001).

CD68 and iNOS are activation marker proteins of M1-like macrophages. Increased CD68 expression was found after CFA induction. Even in CRP -/- rats, increased CD68 expression could easily be determined. iNOS expression was obviously highest in the CFA group compared to the other groups. A small increase was observed in the *C* + *C* group compared to the CRP group, but the value was significantly lower than that in the CFA group (Figures 7(a)–7(h), 7(q), and 7(r)).

RANKL, which induces osteoclast activation and cartilage degeneration in OA, was increased obviously in the CFA group but not that much in the *C* + *C* group compared to the corresponding controls (Figures 7(i)–7(l), and 7(s)). Moreover, a greater increase in TRAP-positive cells, indicating osteoclasts, was observed in the CFA group than in the *C* + *C* group compared to their corresponding control groups (Figures 7(m)–7(p) and 7(t)).

## 4. Discussion

The contradictory results of previous studies have caused uncertainty about the association between CRP expression and OA progression for a long time. Nicholas's study with CRP-deficient mice demonstrated that CRP exerts beneficial effects in CIA by dampening inflammatory responses [27]. Another study showed a protective effect of CRP at the beginning of arthritis using rabbit CRP-transgenic mice [28]. Injection of anti-CRP antibodies attenuated bone erosion and bone resorption and prevented bone loss in rats. In addition, liver-targeted CRP siRNA, which decreased CRP expression in hepatocytes, exerted an effect similar to that of anti-CRP antibodies in Liang et al.'s study [25]. Additionally, CRP signalling was highly active in the synoviocytes from RA patients, and the addition of exogenous CRP could induce synovial inflammation by activating NF-*κ*B signalling [32].

Some researchers attributed these phenomena to intervention at different stages of RA and suggested that CRP may play a protective role during the early stage of RA but exert detrimental effects during active RA [25, 28]. Related studies, which revealed that different conformations of CRP bind to different corresponding receptors and activate different pathways, may partially elucidate both the proinflammatory and anti-inflammatory effects of CRP [33, 34]. Another factor that cannot be ignored is genetic variation. Based on Rhodes et al.'s study, a 232% difference in the CRP levels in RA patients was attributable to genetics alone [35]. Thus, serum CRP levels should be influenced by underlying inflammation together with genetic variation [18]. Large variations in the serum CRP levels in SD rats and DBA/1 mice were detected in Liang et al.'s study, and CRP interference therapy attenuated the bone damage in animals with CIA that exhibited high CRP levels [25]. In addition, the effect of metabolism should be taken into consideration, since human CRP exacerbated OA development in high-fat diet-fed mice [36]. In the study of Yang et al.'s group, who used the same batch of CRP knockout rats as ours, CRP was found to regulate energy balance and glucose homeostasis through leptin's central effect and hypothalamic signalling [29]. These studies indicated that elevated CRP level during inflammation should be the premise for exploring the effect of CRP on OA, which is influenced by inflammation progression, genetic variation, and metabolism.

In the present study, we demonstrated that CFA-induced TMJ inflammation increased the systemic and local CRP expression (Figures 1 and 4). Furthermore, CRP-/- rats exhibited less severe inflammatory symptoms and a lower degree of inflammatory cytokine expression. Even with milder inflammatory manifestations, hyperplastic synovial membranes, thickened discs, diffuse inflammatory cell infiltration, and abundant lipid droplets appeared in the *C* + *C* group, and inappreciable changes were observed in the cartilage (Figures 3–5). These results indicated that controlling highly elevated CRP levels during inflammation should be beneficial, at least for preventing the rapid progression of TMJ-OA to hard tissue.

Related studies demonstrated that the interaction of CRP with its receptor promoted the production of proinflammatory cytokines, leading to amplification of the inflammatory reaction [26, 37]. In CFA-induced TMJ inflammation, the proinflammatory factors IL-1*β* and TNF-*α* were specifically upregulated [11, 12, 38]. By RT-qPCR analysis and IHC staining of the synovial membrane, we found lower levels of IL-1*β* and IL-6 in the *C* + *C* group than in the CFA group (Figure 6). However, IL-2 did not exhibit that obvious decline, which demonstrated that factors other than CRP regulated cytokines profile in this context. In addition, IL-10, which acts as an anti-inflammatory factor, was downregulated in the CFA-induced inflamed TMJ, and its expression increased when CRP was deleted [11]. The combined effects of these inflammatory factors reduced the symptoms in the *C* + *C* group. Thus, in conditions of elevated CRP and TMJ inflammation, CRP affected inflammatory symptoms by modulating the cytokine profile.

M1-like macrophages, which act as the main producers of inflammatory mediators, such as IL-1*β*, IL-6, and TNF-*α*, play a major role in joint inflammation [38, 39]. In contrast, M2 macrophages, which produce IL-10, play an anti-inflammatory role [40]. We found that CD68 and iNOS were highly expressed in the CFA group, which showed M1 macrophage activation. CD68 and iNOS expression levels in the *C* + *C* group were reduced, which explained the decreased inflammatory reactions.

RANKL, as the core component of NF-*κ*B signalling, mediates the synthesis of catabolic factors and activation of osteoclasts, resulting in cartilage degeneration in OA [41]. Degenerative changes were observed only in the cartilage of the CFA group, and more RANKL-positive cells were observed. The numbers of TRAP-positive cells, which represent osteoclasts, were further increased in the CFA group compared with the *C* + *C* group. These results demonstrated that increased CRP levels promoted RANKL expression and contributed to cartilage degeneration. A study on the role of CRP in osteoclastogenesis revealed that CRP neutralized RANKL to inhibit RANKL-induced osteoclastic differentiation, and monomeric CRP (mCRP) promoted this process in the absence of RANKL [33]. When the concentration of mCRP was higher than that of RANKL, inhibition of CRP exerted a protective effect in OA [25]. Similarly, CRP functions as a positive regulator of macrophage activation when it binds to DNA from apoptotic cells. Thus, in addition to the factors mentioned previously (inflammation progression, genetic variation, and metabolism), the exact role of CRP in OA is conformation-dependent and dependent on binding factors.

Given the results of the present study, we concluded that the downregulation of highly elevated CRP levels is beneficial in TMJ-OA and results in milder inflammatory reactions and less cartilage degeneration. This deduction is based on the effects of CRP knockout on the cytokine profile, macrophage activation, and osteoclast differentiation. Since silencing CRP alone could not reverse the inflammatory manifestations, combinations with additional strategies focused on inflammatory factors and the immune response, which require further exploration, are needed to improve treatment outcomes.

## Data Availability

Data available on request to Yao He from e-mail: yaohe@hospital.cqmu.edu.cn.

## Supplementary Materials

Supplementary Materials Supplemental Table: primers used for RT-qPCR.
