# Supplementary material for: C-Reactive Protein Knockout Attenuates Temporomandibular Joint Inflammation in Rats
Source: J Immunol Res. 2022 Jan 10;2022:8613986. doi: 10.1155/2022/8613986 (PMC8763526; doi:10.1155/2022/8613986)
Supplement: Supplementary Materials — Supplemental Table: primers used for RT-qPCR. [file 8613986.f1.pdf]

Supplemental Table. Primers used for RT-qPCR

Gene	Forward	Reverse
**CRP**	ATTTCCCAGTCTGTAAATAAGCAAA	AATGGGAAATGGTAACATATTAATC
**TNF-α**	CATCTTCTCAAAATTCGAGTGACAA	GGGAGTAGACAAGGTACAACCC
**IL-1β**	CAACCAACAAGTGATATTCTCCATG	GATCCACACTCTCCAGCTGCA
**IL-2**	CGGCACCATCCTAAACTGTGA	GGGAGTTGCTTGTGCACTGA
**IL-6**	CACACAGACAGCCACTCACC	AGCTCTGGCTTGTTCCTCAC
**IL-10**	CACTGCTATGTTGCCTGCTCTTAC	GGGTCTGGCTGACTGGGAAG
**β-actin**	AGCTGCGTTTTACACCCTTT	AAGCCATGCCAATGTTGTCT
